# The prevalence of high risk of obstructive sleep apnea in patients with psoriasis

**DOI:** 10.1007/s11325-025-03318-y

**Published:** 2025-04-15

**Authors:** Tanchanok Supajarupan, Wish Banhiran, Chanisada Wongpraparut, Leena Chularojanamontri, Narumol Silpa-archa, Navarat Kasemsuk

**Affiliations:** 1 Department of Otorhinolaryngology, Faculty of Medicine Siriraj Hospital, Mahidol University, Bangkok, Thailand; 2 Department of Dermatology, Faculty of Medicine Siriraj Hospital, Mahidol University, Bangkok, Thailand

**Keywords:** Sleep apnea, Obstructive, Psoriasis, Cross-sectional study, Questionnaire

## Abstract

**Purpose:**

To determine the prevalence of high-risk OSA among people diagnosed with psoriasis and to investigate relationships between the risk of OSA and the characteristics of psoriasis.

**Methods:**

This cross-sectional study was conducted after the approval of the review board during February 2023 and February 2024. Inclusion criteria were psoriasis patients aged ≥ 18 years who visited the dermatologic clinic, Siriraj Hospital, Thailand. Demographic data, anthropometric measurements, underlying conditions, types of psoriasis, Psoriasis Area and Severity Index scores, disease duration, percentage of body surface area involvement, Epworth Sleepiness Scale (ESS), and STOP-Bang questionnaire were collected. Patients who were unable to answer these questionnaires were excluded.

**Results:**

Of the 200 participants (106 men, 94 women), 108 patients (54%) were identified as high-risk for OSA; of them, 70 were men (64.8%) and 38 were women (35.2%). Within this group, statistically significant differences were observed in male (*p* < 0.001), age (*p* = 0.02), and the presence of hypertension, diabetes, and dyslipidemia. Both BMI and an ESS score > 10 were also significantly elevated in the high-risk group (*p* < 0.05). However, no significant correlations were detected between various characteristics of psoriasis and the risk of OSA. Only male sex [adjusted odd ratios (OR) = 3.51], HT (OR = 2.87), and an ESS score > 10 (OR = 4.13), showed statistically significant associations with an increased risk of OSA (*p* < 0.05).

**Conclusion:**

Psoriasis patients had a higher prevalence of high-risk OSA compared to the general Thai population. This underscores the importance of screening individuals with psoriasis, particularly those exhibiting concurrent HT, male sex, and EDS, for OSA.

**Supplementary Information:**

The online version contains supplementary material available at 10.1007/s11325-025-03318-y.

## Introduction

Obstructive sleep apnea (OSA) occurs due to temporary obstruction of the upper airways during sleep, which can negatively affect health, leading to symptoms such as fatigue, headaches, increased daytime sleepiness, decreased productivity at work, and increased risk of accidents. Furthermore, it is associated with various diseases such as hypertension (HT), heart disease, cerebrovascular disease, and depression, and contributes to bed partners [1–4]. A systematic review of the prevalence of OSA found a wide range of prevalence in different studies, ranging from 9 to 38%, with higher rates in men (13–33%) compared to women (6–19%) [5]. In Thailand, a study by Neruntarat et al. found that the prevalence of OSA, defined by an apnea-hypopnea Index (AHI) > 5 events/h, was 11.4%, (15.4% males, 6.3% females) [3]. 

Psoriasis is a chronic inflammatory skin disease characterized by red, thickened skin lesions with scales, resulting from abnormalities of the immune system and proliferation of skin cells. The global prevalence of psoriasis is approximately 0.6–4.8% of the population, affecting both males and females of all ages and exhibiting a familial predisposition with a significant ethno-racial difference in psoriasis prevalence and subtypes [2]. Although its exact cause is unclear, it is believed to involve multiple factors including genetics, immune system abnormalities, and external triggers [6]. Psoriasis is also associated with other diseases such as psoriatic arthritis, inflammatory bowel disease, autoimmune diseases, lymphoma, and metabolic syndrome which contribute to higher risk of OSA [7]. Patients with psoriasis have a higher incidence of metabolic syndrome compared to those without this condition, leading to an increased risk of heart and blood vessel diseases [6–9]. In addition to itching or pain at the lesions and abnormal skin temperature regulation [10], psoriasis is associated with inflammation in various bodily systems. These can increase autonomic nervous system activity and cause arousal or awakenings that alter the quality and quantity of sleep, resulting in insomnia and other sleep disorders.

A previous study showed that patients with OSA have higher levels of IL-6 and TNF-α, which are also found in patients with psoriasis and treatment of OSA patients who had refractory psoriasis with continuous positive airway pressure (CPAP) has been shown to reduce psoriasis severity [11]. Even though the pathophysiological mechanisms are unclear, potential associations between these two conditions have been reported in some literature. For example, the study by Chemello et al. [12] found that, when utilizing the STOP-Bang to measure high-risk OSA, the prevalence of OSA in psoriatic patients was significantly higher than that of OSA in general population, as well as those of Shalom et al. [13] which found the higher prevalence of OSA after reviewing ICD9-CM in psoriatic patients than in their control group. Furthermore, Ilbay et al. [14] reported that 61.40% of psoriatic patients were at high risk of OSA according to the Berlin questionnaire, while Karaca et al. [8] found that 54.5% of patients with psoriasis had polysomnography-confirmed OSA. However, the potential relationships between these two conditions that have been discovered in earlier research are still very limited, particularly in Asians. The primary objective of this study is, therefore, to evaluate the prevalence of high-risk OSA among Thai patients with psoriasis. Secondary objectives are to investigate the correlation between psoriasis severity and the degrees of high risk for OSA, as well as daytime sleepiness.

## Materials and methods

### Study design

The study’s design was cross-sectional and it was conducted after an approval by the Siriraj Institutional Review Board (COA no. Si 122/2023), between February 2023 and February 2024. The study adhered to all principles outlined in the Declaration of Helsinki and its subsequent amendments; all participants gave their written informed consents.

### Research participants

The inclusion criteria were patients age at least 18 years old who had been clinically diagnosed with psoriasis by dermatologists and received treatment at psoriasis clinic, the Department of Dermatology, Siriraj Hospital, Thailand. The exclusion criteria were patients who were unable to answer the questionnaires.

Participants’ information including age, gender, comorbidities, body weight, height. blood pressure, neck circumference, and waist circumference were recorded. Blood test data, especially triglyceride levels, HDL levels, and fasting plasma glucose were gathered to evaluate metabolic syndrome according to the modified Adult Treatment Panel (ATP) III criteria [15]. 

### Assessment of high risk OSA

The STOP-Bang questionnaire was used to assess the risk for OSA. A total score of 3 or more points is considered as high risk of OSA [16, 17]. The higher score represents the higher risk of OSA. In this study, we utilized the Thai version of the STOP-Bang questionnaire with permission [16]. This questionnaire exhibited a sensitivity of 87.3%, specificity of 48.1%, positive predictive value (PPV) of 82.2%, and negative predictive value (NPV) of 52.2% for the diagnosis of OSA.

### Assessment of daytime sleepiness

The Epworth Sleepiness Scale (ESS) is a questionnaire used to assess the level of daytime sleepiness in the two weeks prior to the medical visit. It has been translated into Thai and undergone validation and reliability testing. The scale consists of eight questions, each scored from 0 to 3 points. A score of 0 indicates no chance of dozing or napping, 1 indicates a slight chance (rarely), 2 indicates a moderate chance, and 3 indicates a high chance (regularly). Generally, a total score greater than 10 suggests EDS in patients.

### Assessment of psoriasis characteristics

Data related to psoriasis, including type, duration of the condition, percentage of affected body surface area (%BSA), and the Psoriasis Area and Severity Index (PASI) score were collected. The PASI score was used to assess the severity and extent of psoriatic lesions, ranging from 0 to 72 points. Scores below 5 indicate mild severity, 5–10 indicate moderate severity, and scores exceeding 10 indicate severe severity [18]. 

### Statistical analysis

The qualitative data was reported as numbers and percentages, and the quantitative data was reported as mean and standard deviation (SD), or median and interquartile range. To compare the relationships of the secondary outcomes, unpaired t-tests or Mann-Whitney U-tests were used for quantitative data, and Chi-square tests or Fisher’s exact tests were used for qualitative data. To identify factors associated with an increased risk of developing OSA, both univariate and multivariate analyses were performed. The results were reported as crude and adjusted relative risk/odds ratio (OR) along with 95% confidence intervals (CI), using log-binomial regression or logistic regression. A p-value < 0.05 was considered to be statistically significant. SPSS statistics version 27.0 (SPSS, Inc, an IBM Company, Chicago, Illinois) was utilized.

## Results

A total of 200 patients (106 men and 94 women). were enrolled, with a mean age of 49.8 ± 15.1 years. The metabolic syndrome, which includes HT, diabetes mellitus (DM), and dyslipidemia (DLP), was identified in 80 individuals (40%). The majority of patients had mild degree of psoriasis severity, with plaque type accounting for 88.5% of cases. Table 1 provided more information on the study population’s clinical characteristics and demographics.

**Table 1:** Demographic characteristics of study participants

Demographic data	Results
**Gender**, n **(%)**	
Male Female **Age (years)**, mean ± SD **Anthropometric measurement**, mean ± SD	106 (53.0) 94 (47.0) 49.77 ± 15.10
BMI (kg/m^2^)	26.7 ± 6.2
Neck circumference (cm)	35.0 ± 4.3
Waist circumference (cm)	90.8 ± 15.4
**Underlying diseases**, n (%)	124 (62.0)
Hypertension	81 (40.5)
Diabetes Mellitus	47 (23.5)
Dyslipidemia	68 (34.0)
Cerebrovascular disease	7 (3.5)
**Metabolic syndrome**, (*n* = 183)	80 (43.7)
**Psoriasis type**, n (%)	
Plaque	177 (88.5)
Guttate	5 (2.5)
Pustular	4 (2.0)
Erythrodermic	13 (6.5)
Inverse	1 (0.5)
**Psoriatic Arthritis** n (%)	68 (34.0)
**Psoriasis Severity** (*n* = 192)	
Mild	100 (52.1)
Moderate	45 (23.4)
Severe	43 (22.4)
**Duration (year)**, median [IQR]	10.0 [6.0–20.0]
**PASI score**, median [IQR]	4.6 [2.2–9.4]
**%BSA**, median [IQR]	5.0 [2.0–10.0]

SD, standard deviation; IQR, interquartile range; BMI, Body Mass Index; PASI, Psoriasis Area Severity Index; %BSA, percentage of affected body surface area

**Note** The missing data were eight for the PASI score and psoriasis severity

**Note** The missing data were seventeen for the metabolic syndrome

Of the 200 patients, 108 (54%) were classified as being at high risk for OSA (95% CI 44.3–65.2), with 70 males (64.8%) and 38 females (35.2%). More male and older patients were found in the high-risk OSA group. Table 2 shows that compared to the low-risk OSA groups, the high-risk group had significantly higher BMI, neck circumference, waist circumference, ESS scores, and a higher prevalence of underlying diseases such as DLP, DM, and HT. However, there were no statistically significant differences in psoriatic characteristics between the high- and low-risk groups, or between the severity of psoriasis and ESS scores (Table 3). The results of multivariate regression analysis showed that ESS > 10, HT, and male gender were independently associated with high-risk OSA (Table 4). However, we found no significant association between high-risk OSA and the other aspects of psoriasis, such as types, duration, PASI score, severity, and %BSA.

**Table 2:** Variables comparing patients classified as high risk for obstructive sleep apnea (OSA) based on the STOP-Bang questionnaire and those classified as low risk

Factors	High-risk OSA (*n* = 108)	Low-risk OSA (*n* = 92)	*p*-value
**Gender**			
Male, n (%)	70 (64.8)	36 (39.1)	< 0.001*
Female	38 (35.2)	56 (60.9)	
**Age (years)**, mean ± SD	52.05 ± 14.79	47.10 ± 15.10	0.020*
**Anthropometric measurement**, mean ± SD			
BMI (kg/m^2^)	28.17 ± 7.07	24.95 ± 4.56	< 0.001*
Neck circumference (cm)	36.46 ± 4.32	33.23 ± 3.55	< 0.001*
Waist circumference (cm)	95.14 ± 16.63	85.73 ± 11.95	< 0.001*
**Underlying diseases**, n (%)			
Hypertension	62 (57.4)	19 (20.7)	< 0.001*
Diabetes Mellitus	34 (31.5)	13 (14.1)	0.004*
Dyslipidemia	50 (46.3)	18 (19.6)	< 0.001*
Cerebrovascular disease ^F^	6 (5.6)	1 (1.1)	0.127
**ESS > 10**, n (%)	31 (28.7)	11 (12.0)	0.004*
**Psoriasis type**, n (%) ^F^			
Plaque	95 (88.0)	82 (89.1)	1.000
Guttate	3 (2.8)	2 (2.2)	
Pustular	2 (1.9)	2 (2.2)	
Erythrodermic	7 (6.5)	6 (6.5)	
Inverse	1 (0.9)	0	
**Psoriatic arthritis**, n (%)	34 (31.5)	34 (37.0)	0.415
**Psoriasis Severity**, n (%)			
Mild	60 (58.9)	44 (48.9)	0.128
Moderate	18 (17.6)	27 (30.0)	
Severe	24 (23.5)	19 (21.1)	
**Duration (year)**, median [IQR]	10.5 [5.0-20.8]	10.0 [6.0–18.0]	0.405
**PASI score**, median [IQR]	4.5 [2.5–9.2]	5.1 [1.8–9.5]	0.575
**%BSA**, median [IQR]	5.0 [2.0–10.0]	5.0 [2.0–10.0]	0.970

SD: standard deviation; IQR: interquartile range; BMI: body mass index; PASI: Psoriasis Area Severity Index; %BSA: percentage of affected body surface area; ESS: Epworth Sleepiness Scale

**Note** The missing data were eight for the PASI score and psoriasis severity

* Statistically significant at p-value < 0.05

**Table 3:** Association between psoriasis severity and Epworth sleepiness scale (ESS) score categories

Psoriasis Severity	ESS > 10 (*n* = 37)	ESS ≤ 10 (*n* = 155)	*p*-value
Mild	19 (51.4)	85 (54.8)	0.070
Moderate	5 (13.5)	40 (25.8)	
Severe	13 (35.1)	30 (19.4)	

ESS, Epworth Sleepiness Scale

**Note** The missing data were eight for the psoriasis severity

* Statistically significant at p-value < 0.05 determined by Chi-square test

**Table 4:** Factors associated with high-risk OSA in psoriatic patients

Factors	Crude OR	95%CI	*p*-value	Adjusted OR	95%CI	*p*-value
Age	1.02	1.00–1.04	0.022*	1.03	1.01–1.05	0.058
Male gender	2.86	1.61–5.09	< 0.001*	3.51	1.36–9.05	0.009*
Hypertension	5.18	2.75–9.75	< 0.001*	2.87	1.26–6.55	0.012*
Diabetes Mellitus	2.77	1.37–5.70	0.005*	1.26	0.49–3.25	0.633
Dyslipidemia	3.54	1.87–6.72	< 0.001*	2.05	0.86–4.84	0.103
Cerebrovascular disease	0.18	0.02–1.58	0.124	NA		
BMI	1.09	1.04–1.16	< 0.001*	1.10	0.97–1.24	0.127
Neck circumference	1.22	1.13–1.33	< 0.001*	1.14	0.98–1.33	0.095
Waist circumference	1.04	1.02–1.07	< 0.001*	0.98	0.93–1.03	0.333
ESS > 10	2.97	1.39–6.31	0.005*	4.13	1.66–10.25	0.002*

BMI, Body Mass Index, ESS, Epworth Sleepiness Scale

NA: Not Analyzed due to non-significant univariate results

**Note** Variables with non-significant results in univariate analysis were not included in the multivariate analysis

* Statistically significant at p-value < 0.05 determined by logistic regression

## Discussion

The results of this study revealed that 108 subjects with psoriasis (54%) were found to be at high risk for OSA according to the STOP-Bang (95% CI 44.3–65.2%). This prevalence was substantially higher than the global prevalence of OSA (9–38%) as reported by Senaratna et al. [5] and higher than those in the Thai population (11.4%) as reported by Neruntarat et al. [3] Our findings, however, are consistent with the prevalence of OSA in psoriatic patients and show some similarities with previous reports from Chemello et al. (34.6%), Ilbay et al. (61.4%), Karaca et al. (54.5%), and others in terms of age groups, gender distribution, and PASI score [8, 12, 14, 19, 20]. These findings suggest that there was potentially associations between psoriasis and OSA. However, at present, it remains unclear whether OSA serves as a risk factor for the development of psoriasis or if psoriasis might be a potential predictor of OSA.

Recent advances in understanding the pathogenesis of OSA have highlighted four primary factors including an impaired upper airway collapsibility, low respiratory arousal threshold, high loop gain, and compromised control and function of pharyngeal dilator muscles during sleep [21]. The recurrent hypoxia and sleep disruptions characteristic of OSA may exacerbate psoriasis through mechanisms like oxidative stress and inflammation with some evidence [14] suggesting a potential dose-response relationship between psoriasis severity and OSA risk. On the other hand, psoriasis may disrupt sleep, through itching, pain, and scratching, resulting in arousals, fragmented sleep, instability of respiration, and sympathetic activation, all of which potentially trigger OSA [7, 22, 23]. Psoriasis and OSA also share common features such as the secretion of various mediators, including tumor necrosis factor (TNF)- α, intercellular adhesion molecule (ICAM)-1, E-selectin, and vascular endothelial growth factor (VEGF), which perpetuate inflammatory process [7, 23, 24]. Plausible biological mechanisms, such as chronic inflammation and shared risk factors like obesity and various comorbidities including cardiovascular and metabolic conditions, support the bidirectional cause-effect relationship between psoriasis and OSA [7, 23]. Nevertheless, further long-term studies is still needed.

Statistically significant differences encompassing gender, age, HT, DM, DLP, BMI, neck circumference, and waist circumference were observed between the low-risk OSA group and the high-risk OSA group, consistent with previous studies [12, 14, 25]. The association between psoriasis and obesity or metabolic syndrome as determined by higher BMI were shown in earlier reports [26]. 

The results of this study revealed no statistically significant difference between the high- and low-risk groups of OSA with respect to psoriasis parameters such duration, %BSA, PASI score, and severity, which is consistent with the findings of studies by Chemello et al. [12] and Karaca et al. [8] These results, however, differ from those of the study by Papadavid et al. [9] which reported a statistically significant difference in duration, PASI score, and severity, or the study by Ilbay et al. [14] which found that the high-risk OSA group had a significantly higher mean PASI (*p* = 0.018) and longer mean disease duration than the low-risk group.

The lack of correlation between psoriasis severity (PASI) and OSA risk (STOP-Bang) likely arises from differences in what these measures assess. PASI measures the size of skin lesions but not systemic inflammation or comorbidities, whereas STOP-Bang assesses demographic and physical characteristics such as age, neck circumference, and BMI that may affect OSA risk without regard to the severity of psoriasis. Furthermore, common processes like inflammation may not correlate directly with PASI scores. The limitations of the study, such as its cross-sectional design and dependence on subjective measures, might also possibly contribute to the conflict findings.

There were no statistically significant results regarding the relationships between psoriasis types including psoriatic arthritis and high-risk OSA which is consistent with the report of Papadavid et al. [9] These are divergent from a cohort study by Egeberg et al. [27] which demonstrated an increased risk for OSA among patients with mild and severe psoriasis, as well as those with psoriatic arthritis. There are a few possible explanations for this discrepancy. First, it could have been caused by differences in study design and methods. While Egeberg et al. conducted a cohort study, our analysis was cross-sectional which offered a dissimilar viewpoint. Second, it could have been caused by variations in how high-risk OSA and psoriasis severity were defined and assessed. While our study employed clinical diagnosis and STOP-Bang, the prior study reviewed the diagnosis using ICD-10 codes. And third, the sample size was much larger in Egeberg et al. [27]

In line with the findings of Ilbay et al. [14], the study’s results demonstrated that EDS, as defined by ESS > 10, was a factor associated with high-risk OSA in psoriatic individuals. It is recognized that some cytokines, particularly TNF-α and IL-6 which are frequently seen in psoriasis and OSA [28] could induce sleep and may cause EDS. Thaci et al. found that sleep significantly improved with etanercept treatment, a TNF-α antagonist. However, we did not gather information regarding the treatments of the subjects [29]. Although it did not reach statistical significance, there was a trend toward a relationship between EDS and psoriasis severity. EDS is more common in patients with more severe psoriasis. The insignificant finding observed in this study might be due to the milder degree of disease severity among participants in contrast to a study by Strober et al. [30] where the PASI score was found to be a significant predictor of EDS.

The findings suggest that psoriasis patients, especially males, older individuals, and those with comorbidities like obesity or hypertension, should be screened for OSA using tools like the STOP-Bang questionnaire, as OSA risk is higher in this population regardless of psoriasis severity (PASI). Clinical care should focus on raising awareness of OSA symptoms to ensure early detection and intervention. It is essential to employ multidisciplinary approaches that involve sleep specialists and dermatologists in order to address these overlapping conditions.

This study had potential limitations. First, the STOP-Bang which is a questionnaire open to subjective responses was used to determine the high risk of OSA. Although polysomnography is considered the gold standard for diagnosing OSA, resource constraints precluded its use in this study. Second, due to the cross-sectional design of the study, we were unable to establish causality or determine the direction of the observed associations. Since we lacked a control group, we were unable to compare our findings with a non-psoriatic population. Moreover, there was some missing data so this might be affecting the results of this study.

For future research, it would be beneficial to conduct a longitudinal study, such as a prospective study, to establish a cause-effect relationship between psoriasis and OSA. The study could also involve collecting data on the treatment of psoriasis to determine its impact on the risk of developing OSA. Additionally, using objective measures such as polysomnography for diagnosing OSA would enhance the accuracy of the results.

## Conclusion

The prevalence of high-risk OSA in psoriatic patients was found to be higher than in general population. A significant association was observed between EDS and psoriasis severity. However, there was no significant correlation between psoriasis characteristics and high-risk OSA. Our findings emphasize the importance of screening patients with psoriasis, especially those with underlying HT, male gender, and EDS, for OSA. Given the significant correlation between these factors and high-risk OSA identified in our study, early evaluation and management of OSA in this patient with psoriasis may be warranted to improve overall health outcomes.

## Electronic supplementary material

Below is the link to the electronic supplementary material.

Supplementary Material 1

Supplementary Material 2

## Data Availability

The data that support the findings of this study are not openly available due to reasons of sensitivity and are available from the corresponding author upon reasonable request.
