# Supplementary material for: The prevalence of high risk of obstructive sleep apnea in patients with psoriasis
Source: Sleep Breath. 2025 Apr 15;29(2):160. doi: 10.1007/s11325-025-03318-y (PMC12000173; doi:10.1007/s11325-025-03318-y)
Supplement: Supplementary file 1 — Supplementary Material 1 [file 11325_2025_3318_MOESM1_ESM.pdf]

2 WANGLANG Rd. BANGKOKNOI
BANGKOK 10700

Tel. +66 2419 2667-72
Fax. +66 2411 0162

Siriraj Institutional Review Board

Certificate of Approval

COA no. Si 122/2023

Protocol Title (English)	:	The prevalence of high risk of obstructive sleep apnea in patients with psoriasis
Protocol Title (Thai)	:	ความชุกของผู้มีความเสี่ยงสูงต่อการเป็นโรคหยุดหายใจขณะหลับจากการอุดกั้นในผู้ป่วยโรคสะเก็ดเงิน
SIRB Protocol No.	:	038/2566(IRB1)
Principal Investigator/Affiliation	:	Assoc. Prof.Wish Banhiran M.D. / Department of Oto – Rhino – Laryngology
Research site	:	Faculty of Medicine Siriraj Hospital
Duration of research	:	1 year
Approval date	:	February 13, 2023
Expired date	:	February 12, 2024

This is to certify that Siriraj Institutional Review Board is in full compliance with international guidelines for human research protection such as the Declaration of Helsinki, the Belmont Report, CIOMS Guidelines and the International Conference on Harmonization in Good Clinical Practice (ICH-GCP)

(Prof.Naraporn Prayoonwiwat, M.D.)
Chairperson

15 FEB 2023
date

(Prof.Apichat Asavamongkolkul, M.D.)
Dean of Faculty of Medicine Siriraj Hospital

16 FEB 2023
date

Approval includes :

1. SIRB submission form, Version 1 date February 10, 2023
2. Participant information sheet, Version 2 date February 10, 2023
3. Informed consent form
4. Case record form
5. STOP-Bang Questionnaire
6. The Epworth Sleepiness Scale (ESS)
7. Instruction for obstructive sleep apnea (OSA)
8. Curriculum vitae

หน่วยพิมพ์โรงพยาบาลศิริราช 2341/3,000 แผ่น/ต.ค.57/92667/Mat.10023252/3060/Mo.
