# Supplementary material for: The prevalence of high risk of obstructive sleep apnea in patients with psoriasis
Source: Sleep Breath. 2025 Apr 15;29(2):160. doi: 10.1007/s11325-025-03318-y (PMC12000173; doi:10.1007/s11325-025-03318-y)
Supplement: Supplementary file 2 — Supplementary Material 2 [file 11325_2025_3318_MOESM2_ESM.docx]

11 June 2024

Dear Editor,

# I am a scientific editor working for the Siriraj Hospital Faculty of Medicine, Mahidol University. I edit medical manuscripts on behalf of the Siriraj Medical Research Center (SiMR) and the Siriraj Institute of Clinical Research (SICRES). This letter confirms that I have edited the manuscript entitled ‘The Prevalence of High Risk of Obstructive Sleep Apnea in Patients with Psoriasis’, authored by Dr. Navarat Kasemsuk and colleagues of the Department of Otorhinolaryngology and Department of Dermatology Faculty of Medicine, Siriraj Hospital, Mahidol University, Thailand

Respectfully,

***Mark***

James Mark Simmerman, Ph.D.

Editor and Content Developer

Chiangmai, Thailand

[marksimmerman@protonmail.com](mailto:marksimmerman@protonmail.com)
